# Initiation of Conceptus Elongation Coincides with an Endometrium Basic Fibroblast Growth Factor (FGF2) Protein Increase in Heifers

**DOI:** 10.3390/ijms21051584

**Published:** 2020-02-26

**Authors:** Daniel Chiumia, Katy Schulke, Anna E. Groebner, Nadine Waldschmitt, Horst-Dieter Reichenbach, Valeri Zakhartchenko, Stefan Bauersachs, Susanne E. Ulbrich

**Affiliations:** 1 ETH Zurich, Animal Physiology, Institute of Agricultural Sciences, 8092 Zurich, Switzerland; daniel.chiumia@usys.ethz.ch; 2 Physiology Weihenstephan, Technical University of Munich, 85354 Freising, Germany; katyschulke@yahoo.de (K.S.); annaehennersperger@gmail.com (A.E.G.); nadine.waldschmitt@tum.de (N.W.); 3 Bavarian State Research Center for Agriculture, Institute of Animal Breeding, 85586 Poing, Germany; Horst-Dieter.Reichenbach@lfl.bayern.de; 4 Chair for Molecular Animal Breeding and Biotechnology, Gene Center, Ludwig-Maximilians University, 81377 Munich, Germany; V.Zakhartchenko@gen.vetmed.uni-muenchen.de (V.Z.); stefan.bauersachs@uzh.ch (S.B.); 5 Vetsuisse Faculty Zurich, University of Zurich, Eschikon 27, AgroVet-Strickhof, 8315 Lindau (ZH), Switzerland

**Keywords:** angiogenesis, bovine, embryo, FGF2, interferon-tau

## Abstract

Fibroblast growth factors (FGF) play an important role during embryo development. To date, the role of FGF and the respective receptors (FGFR) during the preimplantation phase in cattle are not fully characterized. We examined FGF1, FGF2, FGFR1, FGFR2, and FGFR3 in cyclic and early pregnant heifers at Days 12, 15, and 18 after insemination (Day 0). Endometrial FGF1 mRNA transcript abundance in heifers varied significantly with respect to the day after insemination, the pregnancy status, and their interaction. The expression was higher in nonpregnant than in pregnant heifers at Day 18. The conceptus transcripts abundance of FGFR2 and FGFR3 were significantly lower at Day 15 than 18. In the endometrium, FGF1 protein abundance significantly decreased from Day 12 onwards and FGF2 protein abundance showed a minor, but a significant increase at Day 15 in comparison to Days 12 and 18. We concluded that the decrease in FGF1 mRNA expression in pregnant heifers at Day 18 points towards a potential contribution of FGF1 in the preimplantation process. Additionally, successful embryo elongation might require a spatiotemporal FGF2 protein increase in the endometrium.

## 1. Introduction

Acidic (FGF1) and basic (FGF2) fibroblast growth factor (FGF) isoforms are well-characterized proangiogenic factors playing important roles in various physiological conditions, including the embryonic development [1,2]. The acidic and basic prototypic heparin-binding FGF were the first to be identified, purified, and sequenced, and, to date, there are more than 22 structurally related members of the FGF family that are described [1,3]. Among all the members of the FGF family, FGF1, FGF2, and FGF9 are the three FGF lacking a signal leader sequence, which plays a critical role in the classical polypeptide secretion pathway [2]. Thus, their mode of action is driven by a nonclassical protein export that still needs to be fully explored [2]. The FGF have a spatial and temporal manner of expression as well as specificity to different FGF receptor (FGFR) isoforms, namely FGFR1 (IIIc), FGFR2 (IIIc), and FGR3 (IIIc) [2,4]. As reviewed previously [5], FGF members are important during the embryonic development period, the organogenesis process for maintenance and growth-related functions of cells, differentiation, survival, and patterning. In mice, FGF1 and/or FGF2 action influences embryo implantation [6] and mice lacking FGR1 and FGFR2 die before embryonic Days 8.5 and 11, respectively [5]. This indicates that the preimplantation embryo is dependent on FGF [5]. In pigs, a pregnancy-specific expression of FGF1 and FGF2 from Days 10 to 14 after insemination (Day 0) has been described [3]. In particular, cytoplasmic and nuclear FGF2 localization increased in the uterine luminal and glandular epithelial cells in pregnant rather than in cycling pigs. Generally, during preimplantation in both humans and animals, a dynamic vascular development is a necessity for the successful establishment of pregnancies [7]. Nevertheless, the preimplantation period in cows is characterized by high embryonic losses resulting in chronic fertility challenges that is highly pronounced in high yielding dairy cows [8,9,10].

The bovine endometrium expresses FGF1 and FGF2, and the modulation of these two FGF isoforms from Days 5 to 16 in both cyclic and pregnant heifers have been reported [11]. In pregnant heifers, the gene expression of FGF1 and FGF2 in the endometrium relate to changes in the expression of their receptors in the conceptus [11]. The bovine uterine epithelium and the conceptus synthesize FGF2, and, between Days 14 and 17, FGF gene expression increases in the elongating conceptuses [12]. This indicates that FGF1 and FGF2 play critical roles during preimplantation in cattle. The pregnancy status itself as well as the concentrations of progesterone (P4) appear not to substantially affect the endometrial tissue gene expression of the ligands (FGF1 and FGF2) in cattle, while the expression of the respective receptors strongly depends on the conceptus developmental stage [11]. However, the involvement of FGF during the period of initiation of the implantation process at around Day 18 in cattle is not known. In cattle, the period from Days 7 to 19 of pregnancy is critical as it comprises the period of conceptus elongation, pregnancy recognition, and the initiation of the implantation process [11]. We aimed at elucidating the modulation of endometrial and embryonic acidic and basic FGF and the associated receptors during the preimplantation phase in cattle.

## 2. Results

### 2.1. Pregnancy Hormone P4 was Affected by Pregancy Status as Expected

The concentration of the steroid hormones progesterone (P4) and estradiol-17β (E2) were analyzed to confirm that all heifers were in the luteal phase of the estrous cycle. In addition, a possible impact on FGF was tested. There was a higher concentration of circulatory P4 in pregnant heifers than nonpregnant heifers at Days 15 (*p* = 0.039) and 18 (*p* = 0.003). The pregnancy status did not affect the concentration of E2 at any of the days assessed (Table 1).

### 2.2. Endometrium and Conceptus FGF and FGFR mRNA Expression was Influenced by the Day of Pregnancy

**Figure 1:**
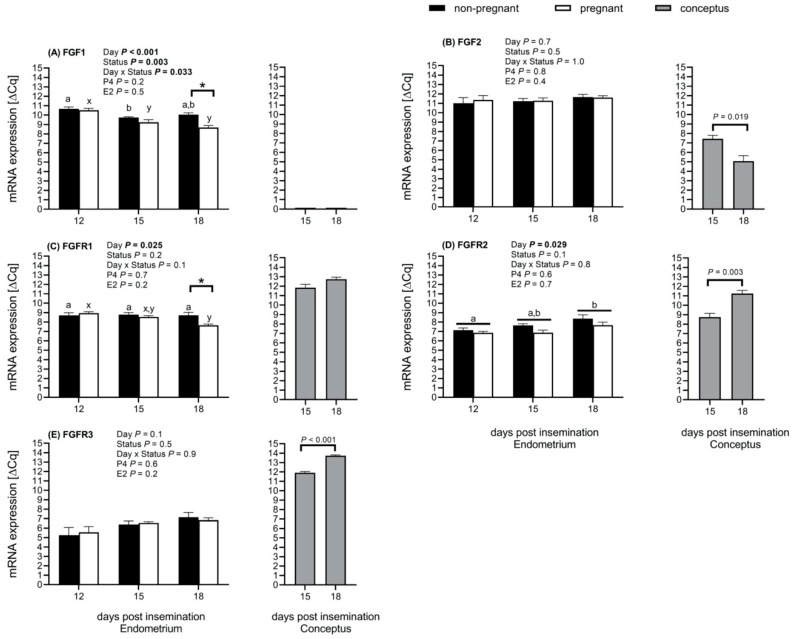
The mRNA was expressed in the endometrium of nonpregnant (*n* = 5 to 8) and pregnant (*n* = 5 to 6) Simmental heifers at Days 12, 15, and 18 post insemination as well as conceptus at Days 15 (*n* = 4 to 8) and 18 (*n* = 4) (insemination = Day 0). (**A**) mRNA expression of fibroblast growth factor 1 (FGF1) and the mRNA transcripts’ abundance was below the detection limit on Days 15 and 18 in the conceptuses; (**B**) FGF2 mRNA expression; (**C**–**E**) mRNA expression of FGF receptors 1 (FGFR1), FGFR2, and FGFR3 (IIIc isoforms). An asterisk (*) indicates significant differences between groups and different letters within nonpregnant (a,b) and pregnant (x,y) heifers indicate significant differences within groups over time (days) (**A**,**B**) and between days (**D**). Abbreviation P4 = progesterone and E2 = estradiol-17β. The results presented as mean delta quantitative cycle (ΔCq) ± standard error of the mean, and high ΔCq represent a high transcript abundance. Differences were considered significant at a 95% confidence interval.

We assessed if the pregnancy status affected the transcripts abundance of the FGF ligands and receptors on both the maternal endometrium and the conceptus. Indeed, we observed that the endometrial FGF1 mRNA expression in heifers depended on the day after insemination (*p* < 0.001), pregnancy status (*p* = 0.003), and the interaction of these two factors (*p* = 0.033). The expression was higher in nonpregnant heifers than pregnant heifers at Day 18 post insemination only (Figure 1A,C).

In pregnant heifers, the mRNA expression of FGF1 decreased from Days 12 to 15 (*p* = 0.003) and remained stable until Day 18. None of the factors that were assessed influenced the mRNA expression of FGF2 and FGFR3 in the endometrium (Figure 1B,E). The mRNA expression of the receptors FGFR1 (*p* = 0.025) and FGFR2 (*p* = 0.029) in the endometrial tissue was influenced by the day of pregnancy (Figure 1C,D). Regardless of the pregnancy status, the endometrial tissue mRNA expression of FGFR2 increased from Days 12 to 18 (*p* = 0.042) (Figure 1D). In the conceptuses, FGF1 mRNA transcripts abundance was below the detection limit on Days 15 and 18 (Figure 1A). In the conceptus tissues, FGF2 mRNA expression decreased from Days 15 to 18 (*p* = 0.019) (Figure 1B). On the other hand, mRNA expression of FGFR2 (*p* = 0.003) and FGFR3 (*p* < 0.001) in the conceptuses increased from Days 15 to 18 (Figure 1D,E).

### 2.3. aFGF and bFGF Protein was Localized in the Endomentrium of both Cyclic and Pregnant Heifers

**Figure 2:**
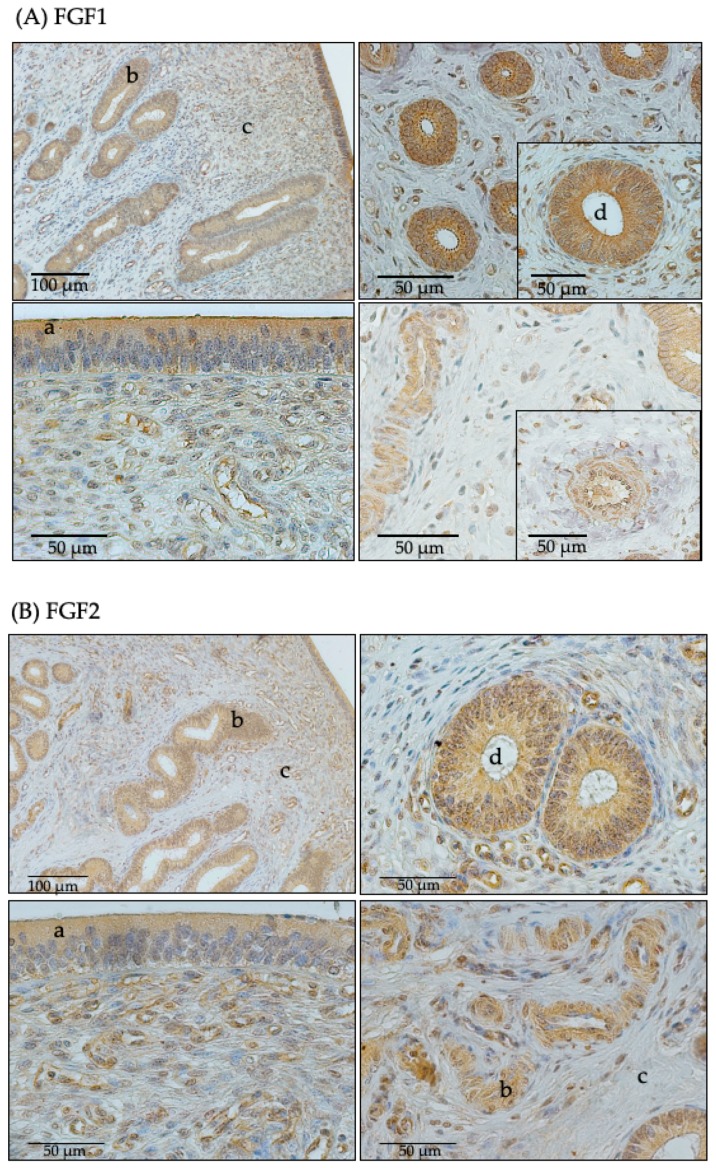

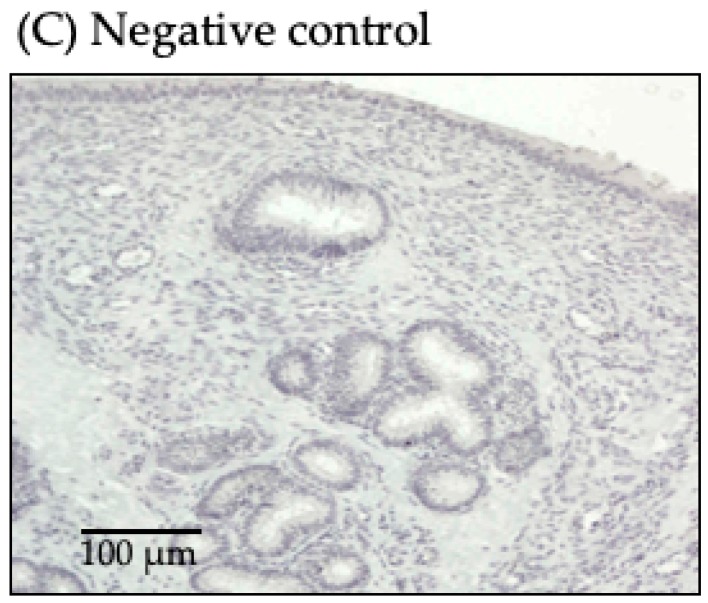
(**A**) Immunohistochemical localization of fibroblast growth factor 1 (FGF1) protein (brown), (**B**) the localization of FGF2 protein, and (**C**) the negative control. Positive staining for FGF1 and FGF2 in the endometrium of Simmental heifers was observed in luminal (**a**) and glandular (**b**) epithelium, in the stromal endometrium (**c**) and in blood vessels (**d**).

We performed an immunohistochemical staining to localize the ligands in the endometrial tissue. The results show that the acidic and basic FGF were expressed in the endometrium of the heifers without differing by pregnancy status. The FGF were primarily localized in the luminal and glandular epithelium as well as in the stroma and blood vessels (Figure 2A,B).

### 2.4. bFGF Protein Abundance Increased at Day 15

**Figure 3:**
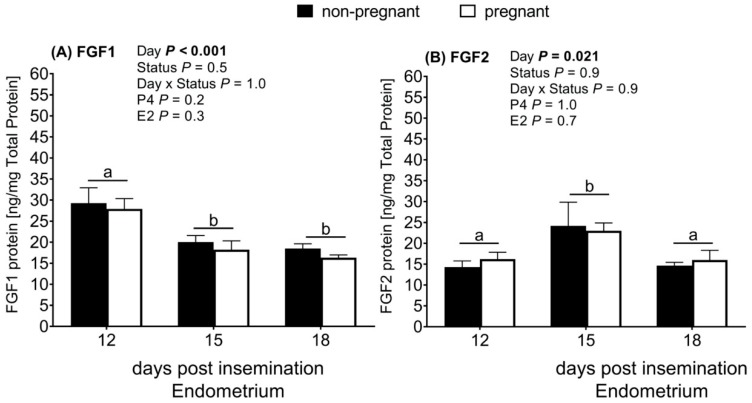
Fibroblast growth factor (FGF) protein abundance in the endometrium of nonpregnant (*n* = 6 to 8) and pregnant (*n* = 5 to 6) Simental heifers after insemination (insemination = Day 0). (**A**) FGF1 protein abundance; (**B**) FGF2 protein abundance. Days with a common letter (a,b) were not significantly different. Differences were considered statistically significant at a 95% confidence interval.

Following the results of the mRNA transcripts’ abundance we observed in the endometrial tissue, we further quantified the protein abundance in the endometrial tissue. The protein abundances of the FGF1 (*p* < 0.001) and FGF2 (*p* = 0.021) in the endometrium depended on the day of pregnancy and there was no significant influence of the pregnancy status (Figure 3A,B).

While the endometrial FGF1 protein abundance decreased from Days 12 to 15 (*p* < 0.001) and remained low until Day 18, the endometrium abundance of FGF2 protein on the other hand increased on Day 15 (*p* = 0.038) from Day 12. On Days 12 and 18, FGF2 protein abundance did not differ (*p* > 0.05).

## 3. Discussion

The regulation of FGF and FGFR in the bovine endometrium and conceptus displays a temporal expression as previously reported [11]. The isoform FGF1 is produced by bovine luminal epithelium in the endometrium during mid-gestation, and Cooke et al. [12] speculated that FGF1 might show a similar gene expression pattern in the endometrial tissue to FGF2 prior to Day 30 of pregnancy. In line with Okumu et al. [11], we conducted analyses regarding the expression of the ligands FGF1, FGF2, and the receptors FGFR-1, -2, and -3 of IIIc isoforms in cyclic and early pregnancy heifers during a comparable time period. Different to a previous study [11], we analyzed the time around the beginning of the implantation process as well. We observed that the mRNA expression during early pregnancy period on Days 12, 15 and 18 after insemination was affected by the day of pregnancy (FGF1, FGFR1 and FGFR2) and status (FGF1) in both cyclic and pregnant heifers. In addition, we observed a significantly lower mRNA expression of FGF1 and the receptor FGFR1 on Day 18 in pregnant heifers when compared to cyclic ones. Our results are in line with Okumu et al. [11] regarding the mRNA expression of the ligands and the receptors until Day 16 of pregnancy. Unique to our study, we have shown that at Day 18 of pregnancy, the mRNA expression was significantly reduced in pregnant heifers. We did not observe significant mRNA expression for the FGF2 and therefore our results are not in agreement with Cooke et al. [12], who speculated that FGF1 and FGF2 may show a similar gene expression pattern before placentation in the endometrial tissue in cattle. We rather observed that the transcript abundance of both FGF1 and FGFR1 at Day 18 of pregnancy significantly decreased in pregnant heifers. As such, we suggest that the observed decrease in mRNA expression in pregnant heifers on Day 18 indicates the potential contribution of the acidic FGF in initiating the implantation process in cattle.

In the conceptuses, we only detected FGF2 mRNA expression that was significantly lower on Day 18 when compared to Day 15 conceptuses. Vice versa, a significantly higher mRNA expression on Day 18 in comparison to Day 15 conceptuses was observed for the receptors FGFR2 and FGFR3. Taken together, the results of endometrial and conceptuses mRNA expression most likely point toward a crosstalk between maternal and conceptus sides. Cooke et al. [12] reported that FGF1 and FGF2 have a similar capacity to induce the pregnancy recognition signal interferon-tau (IFNT) gene expression in a dose-dependent manner in vitro. The pregnancy recognition signal IFNT is produced in trophoblast tissue and acts in a paracrine and autocrine manner to affect gene expression of genes in the endometrium and in the trophectoderm [13,14]. The production of IFNT begins at the late morula stage and early blastocyst stage and then increases from Days 14 and 15 when the conceptus begins to elongate in order to facilitate the embryo–maternal communication [12,14]. Interestingly, we observed a significant decrease in the conceptuses FGF2 gene expression and a concomitant increase in the receptor (FGFR2 and FGFR3) gene expression from Days 15 to 18. The expression of FGF1 when compared to FGF2 was low during the preimplantation period in bovine at Days 11, 14, and 17 previously [12]. Thus, this might explain why in the current study we could not detect the FGF1 mRNA expression on Days 15 and 18 in the conceptuses.

The localization of FGF1 and FGF2 in the luminal epithelium, glandular epithelium, stromal, and blood vessels highlights the importance of these FGF isoforms during the early pregnancy period in cattle. Interestingly, we observed a significant decrease in the endometrium FGF1 protein abundance from Days 12 to 15, which thereafter remained stable until Day 18. Furthermore, the endometrial FGF2 protein abundance significantly increased from Days 12 to 15, which is a period similar to the period in which IFNT production increases. Both the endometrium and the conceptus produce FGF2 [12]. Thus, it is likely that FGF2 might be involved either directly or indirectly in embryo–maternal signaling. Ealy and Yang [15] reported a strong positive correlation between conceptus elongation and IFNT protein expression between Days 12 and 18. Michael et al. [16] showed an increase in IFNT transcripts’ abundance in a bovine trophectoderm cell line following supplementation with FGF2. We suggest that the changes in the endometrial FGF1 and FGF2 protein abundance we observed indicate the possible ongoing embryo-maternal interaction during this critical period of pregnancy recognition initiation in cattle. Considering the FGF2 protein and FGFR1 mRNA expression results, our results are similar to the findings of Lim et al. [17], who showed that FGF2 enhanced the proliferation of the Day 14 bovine endometrial cell lines in a dose-dependent manner via FGFR1 in vitro.

Inferring from a mouse model, FGFR1 null mouse conceptuses have retarded development and the conceptuses die during gastrulation [1]. The embryonic mortality of FGFR2 mouse mutants is mainly due to the lack of the formation of a functional placenta [6]. In porcine, there is a pregnancy-specific expression of FGF1 and FGF2 from Days 10 to 14 after insemination [3]. In humans, FGF2 acts in an autocrine manner in endometrial stroma, and the FGFR1 contributes to an important role in the maturation of the endometrium and the regeneration of the endometrium after a menstrual cycle [18]. Additionally, the neo-vascularization of the endometrium in humans is partially regulated by stroma-derived FGF2 under the influence of E2 and P4 through a paracrine cell-to-cell interaction [19]. In our case, we did not observe a significant influence of P4 and E2 concentrations on FGF ligands or the receptors at neither gene expression nor protein levels. We therefore suggest that the observed temporal increase in the endometrial FGF2 protein abundance might contribute to the regulation of conceptus signaling during embryo–maternal interaction in early pregnancy.

Taken together, the overall observation that neither FGF1 nor FGF2 significantly differed between pregnant and cyclic heifers suggest a general regulation of acidic and basic FGF due to the day post estrus in an anticipation for the attachment of the growing conceptus. The FGF1 and FGF2 might therefore be involved in supporting embryo development during the early pregnancy period in a spatiotemporal manner in cattle.

## 4. Materials and Methods

The animal experiment was conducted in 2007 in Freising, Germany, and was approved by the District Government of Upper Bavaria and were in accordance with the accepted standards of humane animal care in Germany. A trial number was not given and therefore not available.

### 4.1. Animals and Collection of Samples

Cyclic Simmental heifers were cycle synchronized and treated with Estrumate (cloprostenol) (500 mg i.m., Essex Tierarznei, Munich, Germany) as described previously [20]. Briefly, following estrous detection, the heifers were randomly allocated to two treatment groups. Heifers in the pregnant groups (*n* = 5 per group) were inseminated with cryo-preserved semen and the heifers in non-pregnant groups (*n* = 5 to 8 per group) were treated with the supernatant of centrifuged semen from the bull of the same breed. Samples of blood were collected at the time of slaughter for serum P4 and E2 levels quantification. Shortly after slaughter, the uterus was removed and flushed with phosphate-buffered saline (PBS, pH 7.4) solution to recover conceptuses. In pregnant groups, pregnancy was confirmed by the presence of a conceptus in the uterine flush. Endometrial tissue samples ipsilateral to the ovary bearing the corpus luteum were collected for gene expression analysis. Tissue sample aliquots were immediately transferred into tubes containing RNAlater and managed as per manufacturer’s instructions (Thermo Fisher Scientific, Warrington, UK). For the determination of endometrial FGF protein levels, tissue sample aliquots were snap-frozen in liquid nitrogen. The endometrial tissue samples used for the localization of FGF were fixed in Bouin’s solution, washed with ethanol and embedded in paraffin [21]. Samples were stored at −80 °C until further analysis.

### 4.2. Progesterone and Estradiol-17β Analysis

The concentration of P4 (ng/mL) and E2 (pg/mL) in serum was determined using enzyme-linked immunosorbent assay described by Prakash et al. [22]. Intra- and inter-assay coefficients of variation were less than 12%.

### 4.3. Gene Expression Analysis

Total RNA isolation and gene expression analysis were conducted as described previously [20]. Briefly, the total RNA from endometrial and conceptus tissue samples was isolated using a TRIzol reagent (Invitrogen Corporation, Carlsbad, CA, USA), and the quality of the RNA was determined using an Agilent 2100 Bioanalyzer (Agilent Technology, Palo Alto, CA, USA). The RNA amount was spectrophotometrically determined at 260 nm by a Nanodrop 1000 ND-1000 (peqLab Biotechnologie GmbH, Erlangen, Germany). One μg total RNA was reverse transcribed to cDNA in a reaction of 50 µM Random Hexamer Primers (Invitrogen GmbH, Karlsruhe, Germany), 10 mM dNTPs (Fermentas GmbH, St. Leon-Roth, Germany), 5× First Strand Buffer (Promega, Madison, USA) and 200 U M-MLV RT H(-) Enzym (Promega, Wisconsin, USA). The reaction was at 21 °C for 20 min followed by 48 °C for 120 min and 90°C for 2 min. The quantitative PCR reactions were carried out using the LightCycler DNA Master SYBR Green I protocol (Roche Diagnostics GmbH, Mannheim, Germany). Each PCR assay was set up by including a gel verifying the length of the specific amplicon. Then, the specificity of each amplicon was verified by a specific melting point of the PCR product generated after each qPCR run. Table 2 shows a list of primer pairs of all the genes analyzed.

To compute the relative quantification cycle (ΔCq), the mRNA transcript abundance of the respective target genes was normalized against the geometric mean of the reference genes polyubiquitin (UBQ3), Histone (H3F3A), and 18S rRNA. To have high ΔCq representing high mRNA expression, an arbitrary value was added to ΔCq.

### 4.4. Immunohistochemistry of FGF in the Endometrium

Tissue sample cross-sections (4 µm) mounted on Superfrost Plus slides were air dried and incubated at 60° C for 30 min. For immunostaining, sections were deparaffinized twice in chloroform for 15 min following rehydratization in ethanol solutions with decreasing concentrations (100%, 99%, 96%, 90%, 70%, and 50%), each for 2 min. Thereafter, endogenous peroxidase activity was quenched light-proof with 3% H_2_O_2_ in PBS-T for 15 min at room temperature. Antigen presentation was performed in 0.1 M citrate buffer for 15 min at 100° C. An immunostaining procedure was then performed on the sections at room temperature. Non-specific binding sites were blocked with 10% goat serum (Dako Deutschland GmbH, Hamburg, Germany) in PBS-T for 30 min. A specific antibody raised in rabbits against FGF1 [23] and anti-FGF2 [24] kindly provided by Dieter Schams (Physiology Weihenstephan, Freising, Germany) was diluted 1:50 in PBS-T and applied overnight at 4 °C. The samples were washed in PBS-T and incubated with HRP-conjugate secondary antibody (goat anti-rabbit, Sigma-Aldrich Chemie GmbH, München, Germany) for 1h. Immunostaining was performed using 3,3′-diaminobenzidintetrahydrochloride (Sigma-Aldrich Chemie GmbH, München, Germany) in 0.3% H_2_O_2_ PBS for 2 to 10 min. The tissue samples were counterstained with Mayer’s Haemalaun (Carl Roth GmbH, Karlsruhe, Germany).

### 4.5. Endometrium Protein Abundance Determination

Tissue samples (100 mg) were homogenized in 1mL of PBS containing proteinase-inhibitor (Roche Applied Science, Mannheim, Germany) and then centrifuged through a NucleoSpin Filter L (Macherey-Nagel GmbH & Co KG, Düren, Germany). The total concentration of the protein in the respective sample homogenates was determined using a bicinchoninic acid protein standard protocol [25]. Human FGF1 and FGF2 commercial ELISA kits were used to quantify FGF protein levels as per the manufacturer’s instructions (R&D Systems GmbH, Wiesbaden-Nordenstadt, Germany). The respective FGF protein level per sample was normalized with the sample’s total protein concentration determined by bicinchoninic acid assay.

### 4.6. Statistical Analysis

Statistical tests were conducted using IBM SPSS Statistics for Windows, Version 24.0 (Armonk, NY: IBM Corp). To evaluate differences in endometrial gene expression and FGF protein abundance, a linear mixed model was used and the variables in the model were the day of the estrous cycle (Day), pregnancy status (pregnant vs. non-pregnant (Status)), and the interaction between the day of the cycle and pregnancy status (Day x Status). The concentrations of circulating P4 and E2 at slaughter were included in the model as covariates. Bonferroni tests were used in post-hoc analyses. A T-test was used to determine the significant differences in conceptus gene expression between Days 15 and 18. The graphs were produced using GraphPad Prism for Windows, version 7.03 (GraphPad Software, La Jolla, CA, USA). The results were indicated to be significantly different at *p* < 0.05.

## Figures and Tables

**Table 1:** Progesterone and estradiol-17β concentrations at slaughter in the serum of cyclic and early pregnant Simmental heifers after insemination (insemination = Day 0).

Day	Status	Animals	Progesterone [ng/mL]		Estradiol-17β [pg/mL]	
			Mean ± SEM	*p*-Value	Mean ± SEM	*p*-Value
Day 12	nonpregnant	6	8.40 ± 1.02	0.7	3.51 ± 0.61	0.7
	pregnant	5	7.83 ± 0.93		3.23 ± 0.56	
Day 15	nonpregnant	7	6.63 ± 0.86	0.04	1.92 ± 0.52	0.3
	pregnant	6	9.37 ± 0.93		2.81 ± 0.56	
Day 18	nonpregnant	8	6.80 ± 0.80	0.003	1.20 ± 0.48	0.2
	pregnant	5	10.91 ± 1.02		2.32 ± 0.61	

Values presented as mean ± standard error of the mean (SEM). The probability value (*p*-value) for the respective pairs is given.

**Table 2:** Forward (for) and reverse (rev) primer sequences of the reference genes (polyubiquitine, histone and 18S rRNA), the fibroblast growth factor (FGF)-1 and -2 and FGF receptor (FGFR)-1, -2, and -3 isoforms for bovine used in quantitative reverse transcription-PCR.

Primer	Sequence		Fragment Length [bp]	Accession Number
Polyubiquitin	for	AGATCCAGGATAAGGAAGGCAT	198	NM_174133
	rev	GCTCCACCTCCAGGGTGAT		
Histone	for	AGATCCAGGATAAGGAAGGCAT	233	NM_174133
	rev	GCTCCACCTCCAGGGTGAT		
18S rRNA	for	AAGTCTTTGGGTTCCGGG	365	-
	rev	GGACATCTAAGGGCATCACA		
FGF1	for	GCTGAAGGAGAAACCACGAC	317	BC103225
	rev	GTTTTCCTCCAACCTTTCCA		
FGF2	for	GAACGGGGGCTTCTTCCT	288	NM_174056
	rev	CCCAGTTCGTTTCAGTGCC		
FGFR1(IIIc)	for	ACTGCTGGAGTTAATACCACCG	125	NM_001110207
	rev	GCAGAGTGATGGGAGAGTCC		
FGFR2(IIIc)	for	GGTGTTAACACCACGGACAA	139	AJ439896
	rev	CTGGCAGAACTGTCAACCAT		
FGFR3(IIIc)	for	CGCTAACACCACCGACAAG	154	AF368288
	rev	CACCAGCTCCTCCTCAGC		
